# A validated score estimating ambulatory status following radiotherapy of elderly patients for metastatic spinal cord compression

**DOI:** 10.1186/1471-2407-14-589

**Published:** 2014-08-14

**Authors:** Dirk Rades, Jasmin N Evers, Volker Rudat, Amira Bajrovic, Johann H Karstens, Steven E Schild

**Affiliations:** Department of Radiation Oncology, University of Lübeck, Ratzeburger Allee 160, D-23538 Lübeck, Germany; Department of Radiation Oncology, Saad Specialist Hospital, Al-Khobar, Saudi Arabia; Department of Radiation Oncology, University Medical Center Hamburg-Eppendorf, Hamburg, Germany; Department of Radiation Oncology, Hannover Medical University, Hannover, Germany; Department of Radiation Oncology, Mayo Clinic, Scottsdale, AZ USA

**Keywords:** MSCC, Elderly patients, Ambulatory status, Prognostic factors, Score

## Abstract

**Background:**

This study was performed to develop a validated score predicting ambulatory status after radiotherapy (RT) alone for metastatic spinal cord compression (MSCC) in elderly patients.

**Methods:**

1,129 elderly patients (≥65 years) were assigned to the test (N = 565) or validation group (N = 564). In the test group, nine pre-treatment factors (age, gender, tumor type, number of involved vertebrae, pre-RT ambulatory status, other bone metastases, visceral metastases, interval cancer diagnosis to RT, time developing motor deficits) and fractionation regimen were investigated. Factors significantly associated with post-RT ambulatory status on multivariate analysis were included in the score. The score for each factor was determined by dividing the post-RT ambulatory rate at 1 month (%) by 10. The total score represented the sum of these scores.

**Results:**

In the multivariate analysis of the test group, age, primary tumor type, pre-RT ambulatory status, visceral metastases, and time developing motor deficits were significantly associated with post-RT ambulatory status. Total scores were 19 to 41 points. In the test group, post-RT ambulatory rates were 5% for 19-25 points, 35% for 26-30 points, 80% for 31-34 points, and 98% for 35-41 points (p < 0.001). 6-month survival rates were 11%, 21%, 59% and 76%, respectively. In the validation group, post-RT ambulatory rates were 4%, 33%, 77% and 98%, respectively (p < 0.001).

**Conclusions:**

Patients achieving 19-25 points had very poor functional outcomes and survival, and may receive single-fraction RT for pain relief. Selected patients with 26-34 points may benefit from additional surgery. Patients achieving ≥35 points achieved favorable results after RT alone.

## Background

Personalized treatment has been studied more during recent years, particularly in palliative situations such as metastatic spinal cord compression (MSCC). Radiotherapy (RT) alone is the most commonly administered treatment for MSCC world wide. Maintaining or regaining ambulatory function is very important for patients developing MSCC. A randomized trial has suggested that selected patients benefit from upfront decompressive surgery in addition to RT in terms of higher post-treatment ambulatory rates when compared to RT alone [1]. However, many patients, particularly elderly patients, may not be able to withstand a neurosurgical intervention, which is associated with a rate of major complications of >10% even in younger patients [1–4]. Therefore, it appears reasonable to develop an instrument that allows the estimation of the ambulatory status after RT alone in order to better identify patients who benefit from upfront surgery and those who may not need it.

This study was initiated in order to develop a validated tool that helps predict the probability of being ambulatory after RT alone specifically for elderly patients (65 years or older). Elderly patients should be regarded a separate group of patients. The course of their disease and the ability to tolerate aggressive treatments such as spinal surgery are generally different than in younger patients.

## Methods

A total of 1,129 elderly (age ≥65 years) patients treated with RT alone for MSCC between 1995 and 2010 were included in this retrospective study. In this study, “elderly” has been defined according to the homepage of the world health organization (WHO), where it is stated that “most developed world countries have accepted the chronological age of 65 years as a definition of 'elderly' or older person” [5]. In addition, Orimo et al. reported that conventionally, “elderly” has been defined as a chronological age of 65 years old or older, while those from 65 through 74 years old are referred to as “early elderly” and those over 75 years old as “late elderly” [6].

Further inclusion criteria included motor deficits of the lower extremities caused by MSCC, no prior treatment to the involved parts of the spinal cord, and administration of corticosteroids. The data were obtained from the patients themselves, their treating physicians, and the patients’ hospital charts. The study has been approved by the ethics committee of the University of Lübeck. The treatment volumes encompassed one normal vertebra above and below the involved vertebrae. Of the entire cohort, 169 patients (14%) had multi-level involvement by MSCC resulting in more than one treatment volume. Prior to RT the patients were generally presented to a surgeon to discuss the option of decompressive surgery. Patients with MSCC due to vertebral fracture with bony impingement of the spinal cord or nerve roots were not included in this study, since these patients were clear candidates for surgery. In this study patients, who received 1×8 Gy were also included, since several studies have demonstrated that 1×8 Gy is similarly effective compared to fractionated regimens with respect to improvement of motor function and ambulatory status [7–9].

The patients were randomly assigned to the test group (N = 565) or the validation group (N = 564). Patient characteristics were not significantly different between these groups as demonstrated in Table 1. The comparisons of both groups with respect to the distribution of the patient characteristics were performed using the Chi-square test.

**Table 1:** **Patient characteristics of the test group and the validation group**

	Test group n patients (%)	Validation group n patients (%)	p-value
**Age**			0.76
65-70 years	230 (41)	217 (39)	
71-80 years	266 (47)	283 (50)	
≥ 81 years	69 (12)	64 (11)	
**Gender**			0.89
Female	197 (35)	201 (36)	
Male	368 (65)	363 (64)	
**Type of primary tumor**			0.91
Breast cancer	89 (16)	106 (19)	
Prostate cancer	150 (27)	159 (28)	
Myeloma/lymphoma	51 (9)	50 (9)	
Lung cancer	112 (20)	108 (19)	
Cancer of unknown primary	44 (8)	45 (8)	
Renal cell carcinoma	34 (6)	32 (6)	
Colorectal cancer	28 (5)	25 (4)	
Other tumors	57 (10)	39 (7)	
**Number of involved vertebrae**			0.78
1-2	235 (42)	219 (39)	
3-4	191 (34)	196 (35)	
≥ 5	139 (25)	149 (26)	
**Ambulatory status prior to RT**			0.84
Not Ambulatory	234 (41)	228 (40)	
Ambulatory	331 (59)	336 (60)	
**Other bone metastases**			0.99
No	229 (41)	229 (41)	
Yes	336 (59)	335 (59)	
**Visceral metastases**			0.29
No	319 (56)	347 (62)	
Yes	246 (44)	217 (38)	
**Interval from cancer diagnosis to RT of MSCC**			0.67
≤ 15 months	290 (51)	302 (54)	
> 15 months	275 (49)	262 (46)	
**Time developing motor deficits**			0.47
1-7 days	175 (31)	193 (34)	
8-14 days	140 (25)	150 (27)	
> 14 days	250 (44)	221 (39)	
**Radiation regimen**			0.99
1 × 8 Gy	96 (17)	95 (17)	
5 × 4 Gy	154 (27)	157 (28)	
10 × 3 Gy	151 (27)	153 (27)	
15 × 2.5 Gy	64 (11)	67 (12)	
20 × 2 Gy	100 (18)	92 (16)	

In the test group, nine potential prognostic factors were investigated including age (65-70 vs. 71-80 vs. ≥81 years), gender, primary tumor (breast cancer vs. prostate cancer vs. myeloma/lymphoma vs. lung cancer vs. cancer of unknown primary vs. renal cell carcinoma vs. colorectal cancer vs. other tumors), number of involved vertebrae (1-2 vs. 3-4 vs. ≥5), ambulatory status prior to RT (not ambulatory vs. ambulatory), other bone metastases prior to RT (no vs. yes), visceral metastases (extra-spinal non-osseous metastases) prior to RT (no vs. yes), interval between first diagnosis of cancer and RT of MSCC (≤15 vs. >15 months), and time of developing motor deficits prior to RT (1-7 vs. 8-14 vs. >14 days). In addition to these pre-treatment factors, the fractionation regimen has been evaluated (1×8 Gy vs. 5×4 Gy vs. 10×3 Gy vs. 15×2.5 Gy vs. 20×2 Gy). The ECOG performance status was not analyzed, since it was directly related to the pre-treatment ambulatory status. The vast majority of ambulatory patients have ECOG-PS 2, and patients who are not ambulatory have ECOG-PS 3 or 4. In the test group, 252 patients (45%) had ECOG-PS 2, 273 patients (48%) had ECOG-PS 3 and 40 patients (7%) had ECOG-PS 4. In the validation group, 258 patients (46%) had ECOG-PS 2, 271 patients (48%) had ECOG-PS 3 and 35 patients (6%) had ECOG-PS 4.

The potential prognostic factors have been included in a multivariate analysis performed with a logistic regression and the backward stepwise (likelihood ratio) method. The prognostic factors that were significant in the multivariate analysis of the test group were included in the score. The score for each significant factor was obtained by dividing the post-treatment (i.e. 1 month following RT) ambulatory rate (given in%) by 10. The total score represented the sum of the scores for each significant factor. Based on the total scores, four prognostic groups were formed. In order to test the reproducibility of the score, each of the four prognostic groups of the test group was compared to the corresponding prognostic group of the validation group with the Chi-square test.

## Results

**Figure 1:**
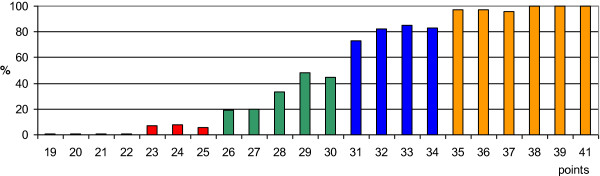
**Test group: the total scores (19 to 41 points) in relation to the ambulatory rate at 1 month following RT (in%).** Four prognostic groups were designed: group A (19-25 points, red columns), group B (26-30 points, green columns), group C (31-34 points, blue columns), and group D (35-41 points, orange columns).

In the multivariate analysis of the test group, ambulatory status at 1 month following RT was significantly associated with age (p = 0.004), visceral metastases (p = 0.017), type of primary tumor (p = 0.002), time developing motor deficits prior to RT (p < 0.001), and pre-RT ambulatory status (p < 0.001). The post-RT ambulatory rates related to the potential prognostic factors, the p-values obtained from the multivariate analysis of the test group, and the corresponding score for each of the five significant prognostic factors are given in Table 2. Total scores ranged from 19 to 41 points (Figure 1).

**Table 2:** **Test group: Ambulatory rates 1 month following RT and the corresponding scoring points**

	post-RT ambulatory rate (%)	p-value	Scoring points
**Age**			
65-70 years (n = 230)	70		*7*
71-80 years (n = 266)	65		*7*
≥ 81 years (n = 69)	48	*0.004*	*5*
**Gender**			
Female (n = 197)	73		
Male (n = 368)	61	0.15	
**Type of primary tumor**			
Breast cancer (n = 89)	85		*9*
Prostate cancer (n = 150)	61		*6*
Myeloma/lymphoma (n = 51)	88		*9*
Lung cancer (n = 112)	66		*7*
Cancer of unknown primary (n = 44)	36		*4*
Renal cell carcinoma (n = 34)	68		*7*
Colorectal cancer (n = 28)	39		*4*
Other tumors (n = 57)	54	*0.002*	*5*
**Number of involved vertebrae**			
1-2 (n = 235)	70		
3-4 (n = 191)	64		
≥ 5 (n = 139)	58	0.28	
**Ambulatory status prior to RT**			
Not Ambulatory (n = 234)	24		*2*
Ambulatory (n = 331)	94	*<0.001*	*9*
**Other bone metastases**			
No (n = 229)	72		
Yes (n = 336)	60	0.64	
**Visceral metastases**			
No (n = 319)	74		*7*
Yes (n = 246)	53	*0.017*	*5*
**Interval from cancer diagnosis to RT of MSCC**			
≤ 15 months (n = 290)	58		
> 15 months (n = 275)	73	0.18	
**Time developing motor deficits**			
1-7 days (n = 175)	30		**3**
8-14 days (n = 140)	70		*7*
> 14 days (n = 250)	87	*<0.001*	*9*
**Radiation regimen**			
1 × 8 Gy (n = 96)	68		
5 × 4 Gy (n = 154)	60		
10 × 3 Gy (n = 151)	60		
15 × 2.5 Gy (n = 64)	66		
20 × 2 Gy (n = 100)	77	0.71

Based on the total scores, the patients were assigned to four prognostic groups, 19-25 points (group A, n = 106), 26-30 points (group B, n = 110), 31-34 points (group C, n = 99), and 35-41 points (group D, n = 250). The ambulatory rates at 1 month following RT were 5%, 35%, 80% and 98%, respectively (p < 0.001, Chi-square test). In group D, the post-RT ambulatory rates were 99% (228/231) at 3 months, 99% (195/196) at 6 months, and 79% (15/19) at 12 months following RT. The 6-month survival rates were 11% in group A, 21% in group B, 59% in group C, and 76% in group D, respectively.

In the validation group, the ambulatory rates at 1 month following RT were 4% in group A, 33% in group B, 77% in group C, and 98% in group D, respectively (p < 0.001, Chi-square test). Each of the groups A to D of the validation group was compared to each of the corresponding groups A to D of the test group with respect to the ambulatory rates at 1 month following RT. The p-values were p = 0.94 for groups A, p = 0.67 for groups B, p = 0.89 for groups C, and p = 0.96 for groups D, respectively.

## Discussion

Personalized treatment has gained importance in palliative oncology and radiation oncology during recent years including prognostic scores [10, 11]. A particular focus has been placed on elderly patients usually defined as 65 years or older, since the proportion of this group of patients in oncology has grown considerably [5, 6]. About 70% of all cancer deaths occur in this age group [12]. The course of the cancer disease in elderly patients is often different from that in younger patients. Moreover, elderly patients may not tolerate or withstand aggressive treatment approaches. Therefore, an over-treatment should be avoided particularly in a palliative situation such as MSCC. Since the mean age of the population in Western countries is increasing, a patient’s performance status and comorbidity must be taken into account in addition to the numeric age. Patients older than 65 years who have a very good performance status and little comorbidity may be treated more aggressively like younger patients.

The majority of MSCC patients are treated with RT alone. However, a small randomized trial of 101 patients revealed that selected patients benefit from upfront decompressive surgery in addition to RT [1]. Since this study was published in 2005, decompressive surgery has seen a “boom” in some countries, particularly in Germany. However, spinal surgery entails significant risks such as wound infections requiring a second surgery, extensive bleeding, postoperative pneumonia, and pulmonary embolism, which occur in more than 10% of the patients [1–4]. Therefore, spinal surgery may be omitted, if reasonably possible. This may be particularly true for elderly patients who have a higher risk of experiencing surgery or anesthesia related complications. In general, surgery for MSCC should be proposed for selected patients, i.e. if there are diagnostic doubts, if stabilization of the vertebral column is required, in case of (impending) sphincter dysfunction, in case of deterioration of motor function during RT, after previous longer-course RT, or if collapse of the vertebral body causes bone impingement on the spinal cord or the nerve roots. According to the study of Patchell et al., decompressive surgery provides also benefit for patients with a relatively good performance status and a relatively survival prognosis [1]. If decompressive surgery is administered, it should include stabilization of the involved vertebra; a simple laminectomy has to be avoided [1–4]. Decompressive surgery should not be used in patients with a Karnofsky performance score of <70, an estimated survival time of less than three months, involvement of more than one spinal segment and very radiosensitive tumors such as myeloma, lymphoma and germ cell tumors [1].

In the present study, or scoring system has been developed to help estimate the probability for elderly patients to be ambulatory following RT alone for MSCC. There is a lack of data regarding post-RT ambulatory status in elderly patients developing MSCC, which was not evaluated in our previous matched-pair analysis comparing surgery plus RT and RT alone [4]. In the multivariate analysis of the present study, five prognostic factors were significantly associated with post-RT ambulatory status. These factors were age, type of primary tumor, pre-RT ambulatory status, visceral metastases, and the time developing motor deficits.

In contrast to these five factors, the radiation regimen was not associated with post-radiotherapy ambulatory status. This finding agrees with previous studies. In two randomized trials from Italy, 1×8 Gy was as effective as 2×8 Gy or a split-course regimen (3×5 Gy plus another 5×3 Gy after a week rest) [8, 9]. Furthermore, in a prospective study and a large retrospective study from Germany, post-treatment functional outcome was similar after short-course RT such as 1×8 Gy and 5×4 Gy when compared to longer-course RT programs such as 10×3 Gy, 15×2.5 Gy and 20×2 Gy [7, 13, 14]. In another prospective study, 10×3 Gy and 20×2 Gy resulted in similar functional outcomes [15].

Six years ago, we presented an ambulatory score based on the data of patients with MSCC of any age [16]. In that score also the interval between the first diagnosis of cancer and RT of MSCC was an independent prognostic factor, but did not play a prognostic role in elderly patients in the current study. This finding supports the concept that elderly patients differ from younger patients and the need for a separate score for the elderly patients.

In the present study, four prognostic groups were defined. The ambulatory rates at 1 month following RT were quite divergent ranging from only 5% to 98%. In addition, the 6-month survival rates of the four groups differed considerably ranging from 11% to 76%. Patients of group A (19-25 points) had both a very low post-RT ambulatory probability and a very poor survival prognosis. RT alone is not successful regarding the functional outcome. These results may be improved with decompressive surgery. However, considering the poor expected survival, surgery may not be a valuable addition. This is supported by the fact that patients with a survival prognosis of less than three months were not included in the randomized trial of Patchell et al. [1]. Also in patients of group B (26-30 points) and group C (31-34 points), the results of RT alone are not optimal and may be improved with the addition of decompressive surgery. Such an approach appears more reasonable for these two groups, since the survival prognoses are better than for group A patients. Patients of group D (35-41 points) showed very good results after RT alone with 98% of patients being ambulatory and 76% of patients living 6 months or longer. It may be questioned whether these patients do need additional surgery? In the current study, the ambulatory rates of group D patients were 99%, 99% and 79% after 3 months, 6 months and 12 months, respectively, following RT. These findings suggest that one may not need to perform additional surgery in group D patients. However, the data included in the current study were retrospectively evaluated.

For validation of this new score, the four prognostic groups A-D of the test group were each compared to the corresponding groups A-D of the validation group. The post-RT ambulatory rates of the four groups of the validation group were very similar to post-RT ambulatory rates of the four groups of the test group. This finding demonstrates that the score is valid and reproducible.

## Conclusions

Patients achieving 19-25 points (group A) had both poor functional outcomes and poor survival. Therefore, these patients may be considered for best supportive care or single-fraction RT if the patients experience pain. Patients with 26-34 points (groups B and C) may benefit from additional surgery, since the functional results of RT alone are not optimal and the patients live longer than those of group A. Patients achieving ≥35 points (group D) had very good results after RT alone. Thus, the group D patients may not require surgery. These findings should ideally be re-evaluated in a prospective study.
